# Regenerative Translation of Human Blood-Vessel-Derived MSC Precursors

**DOI:** 10.1155/2015/375187

**Published:** 2015-07-26

**Authors:** William C. W. Chen, Bruno Péault, Johnny Huard

**Affiliations:** ^1^Department of Bioengineering, University of Pittsburgh, Pittsburgh, PA 15260, USA; ^2^Department of Orthopedic Surgery, University of Pittsburgh Medical Center, Pittsburgh, PA 15213, USA; ^3^Stem Cell Research Center, University of Pittsburgh, Pittsburgh, PA 15219, USA; ^4^Research Laboratory of Electronics and Department of Biological Engineering, Massachusetts Institute of Technology, Cambridge, MA 02139, USA; ^5^Center for Cardiovascular Science, Queen's Medical Research Institute, Edinburgh EH16 4TJ, UK; ^6^MRC Centre for Regenerative Medicine, University of Edinburgh, Edinburgh EH16 4UU, UK; ^7^UCLA Orthopaedic Hospital, Department of Orthopaedic Surgery and David Geffen School of Medicine, University of California at Los Angeles, Los Angeles, CA 90095, USA; ^8^McGowan Institute for Regenerative Medicine, University of Pittsburgh, Pittsburgh, PA 15219, USA; ^9^Medical School and Regenerative and Translational Medicine Institute, University of Texas Health Science Center at Houston, Houston, TX 77030, USA

## Abstract

Mesenchymal stem/stromal cells (MSCs) represent a promising adult progenitor cell source for tissue repair and regeneration. Their mysterious identity *in situ* has gradually been unveiled by the accumulating evidence indicating an association between adult multipotent stem/progenitor cells and vascular/perivascular niches. Using immunohistochemistry and fluorescence-activated cell sorting, we and other groups have prospectively identified and purified subpopulations of multipotent precursor cells associated with the blood vessels within multiple human organs. The three precursor subsets, myogenic endothelial cells (MECs), pericytes (PCs), and adventitial cells (ACs), are located, respectively, in the three structural tiers of typical blood vessels: intima, media, and adventitia. MECs, PCs, and ACs have been extensively characterized in prior studies and are currently under investigation for their therapeutic potentials in preclinical animal models. In this review, we will briefly discuss the identification, isolation, and characterization of these human blood-vessel-derived stem cells (hBVSCs) and summarize the current status of regenerative applications of hBVSC subsets.

## 1. Introduction

Adult multipotent stem/progenitor cells are promising cell sources for tissue repair and regeneration because of their self-renewal, differentiation capacity, and secretion of trophic factors [1]. Though developmentally not as versatile as embryonic stem cells (ESCs) or induced pluripotent stem cells (iPSCs), adult stem/progenitor cells represent a more clinically relevant cell source for regenerative medicine due to less ethical and/or safety issues [2]. In particular, mesenchymal stem/stromal cells (MSCs) and MSC-like multilineage precursor cells, including adipose-derived stem cells (ADSCs), mesoangioblasts, and multipotent adult progenitor cells (MAPCs), have attracted significant clinical attentions, largely owing to their accessibility as well as the robust trophic and immunosuppressive functions.

It has been more than a decade since the first discovery of MSCs and similar precursor cells in human bone marrow (BM), adipose, placenta, and many other tissues [3–5]. Typical MSCs are plastic-adherent and expressing cell surface markers such as CD29 (integrin-*β*1), CD44 (hyaluronic acid receptor), CD73 (ecto-5′-nucleotidase), CD90 (Thy-1), CD105 (endoglin) but negative for CD14 (myeloid cell-specific leucine-rich glycoprotein), CD31 (PECAM-1), CD34 (hematopoietic/stem/endothelial cell marker), and CD45 (pan-leukocyte marker), in addition to the capacity of differentiating into common mesodermal cell lineages including osteoblasts, chondroblasts, and adipocytes. However, BM titers of human MSCs decline significantly with age [6]. Recently, clonally derived MSCs injected intra-arterially have been shown to selectively engraft at perivascular locations in the BM sinusoids/microvessels following a localized radiation injury in the mouse hindlimb [7]. Engrafted MSCs can not only proliferate locally in the long-term but also be serially transplanted into the secondary host while maintaining similar homing and engraftment efficiency. These results imply that BM can be therapeutically exploited as a renewable source for exogenous MSC transplantation.

Despite the extensive investigation, the native identity of MSCs has long been obscured by the retrospective identification method in culture. Recently, there is increasing evidence indicating the relationship between tissue-specific stem/progenitor cells and vascular/perivascular niches [8, 9] as well as the presence of multipotent stem cells of vascular origin [10–12]. Sacchetti et al. demonstrated that CD146+ subendothelial stromal cells residing on the BM sinusoidal wall not only self-renew and display vascular mural cell features but also form bone and establish the hematopoietic microenvironment [13]. Consequently, a hypothesis that blood vessels throughout the human body serve as a reservoir of multipotent precursor cells has been formulated [14, 15].

Using immunohistochemical assays and fluorescence-activated cell sorting (FACS), we and other researchers have prospectively identified and purified three populations of multipotent precursor cells from human blood vessels: myogenic endothelial cells (MECs), pericytes (PCs), and adventitial cells (ACs). Collectively named human blood-vessel-derived stem cells (hBVSCs), these subpopulations of hBVSCs can be isolated from blood vessels within human skeletal muscle, with each expressing a unique profile of cell surface antigens. PCs and ACs have also been isolated from several other human tissues such as fat and lung [16–19]. MECs, PCs, and ACs all possess common mesodermal multipotency and display typical MSC markers, suggesting their contributions to the heterogeneous MSC entity.

## 2. Native Distribution of Human Blood-Vessel-Derived Stem Cells

Human blood vessels typically consist of three definitive structural tiers: tunica intima, tunica media, and tunica adventitia [20]. Tunica intima is the innermost layer of a blood vessel and primarily composed of endothelial cells (ECs), supported by an abluminal layer of elastic fibers. Tunica media contains multiple layers of smooth muscle cells (SMCs) while tunica adventitia, the outermost layer, consists of extracellular matrix (ECM), fibroblast-like stromal cells, and vasa vasorum. At the microvessel (arterioles and venules) and capillary level, the construction of vessels is reduced to only endothelial cells and surrounding vascular stromal cells (VSCs), that is, pericytes. The EC-to-PC ratio ranges from 100 : 1 to 1 : 1 while PC coverage of abluminal EC surface varies from 10% to 50%, depending on the tissue origin [21]. Increasing evidence indicated the existence of pericyte-like cells in normal intima, suggesting a heterogeneous PC network within blood vessels of all sizes [22, 23]. Moreover, comparative characterization studies have demonstrated differences in expression of cell lineage markers, developmental potentials, and angiogenic capacity among human VSCs derived from the walls of blood vessels of different sizes (artery, vein, and microvessel) [24]. These results imply innate differences between VSCs residing in different vascular structural tiers and tissue-dependent divergence of VSCs.

Developmentally myogenic cells (MCs) and ECs of the vertebrate limb may derive from a common somitic precursor [25]. Notch signaling has recently been shown to play an important role in the selection of endothelial versus myogenic cell fate in multipotent somitic Pax3+ cells before their migration to the limbs during embryonic development [26]. Previous studies showed that cells coexpressing MC and EC markers reside within the interstitial space of skeletal muscle and possibly contribute to postnatal muscular development [27]. Consequently we hypothesized that an intermediate cell type coexpressing MC and EC markers exists within the interstitium of postnatal skeletal muscle, presumably associated with blood vessels and potentially multipotent. Indeed, immunohistochemistry revealed that a rare subset of myogenic precursor cells coexpresses MC and EC markers at the microvascular level [28]. These myogenic endothelial cells (MECs) not only express MC markers including Pax7 and CD56 but also display EC markers including CD34, CD144 (VE-cadherin), von Willebrand factor (vWF), and* Ulex europaeus* agglutinin-1 (UEA-1) [28].

Pericytes (PCs) are commonly regarded as a structural component of small blood vessels that regulate vascular contractility, stability, and integrity [29–31]. PCs also modulate EC proliferation/vascular remodeling and are involved in specialized vascular functions including blood-brain barrier and renal tubulovesicular coordination as well as several pathological conditions [21, 23, 32–35]. However, this particular cell population has not been well defined in most of the human organs due to a lack of representative cell marker(s). We previously described microvascular PCs in multiple human tissues based on robust expression of CD146 (Mel-CAM), NG2 (chondroitin sulphate), platelet-derived growth factor receptor-beta (PDGFR*β*), and the absence of myogenic (CD56), hematopoietic (CD45), and endothelial cell surface markers (CD31, CD34, CD144, and vWF) [16]. Alkaline phosphatase (ALP) is another marker used to typify PCs in human skeletal muscle [36, 37]. Alpha-smooth muscle actin (*α*-SMA), on the other hand, can be detected in PCs encircling arterioles and venules but not in those surrounding most capillaries [16]. PCs* in situ* also express classic MSC markers: CD44, CD73, CD90, and CD105 [16].

Adventitial cells (ACs) have been perceived as fibroblast-like cells producing adventitial ECM, a loose structural element enclosing media of arteries and veins. Recent studies indicated that CD34 identifies 2 concentric rings of cells residing in intima and adventitia, respectively [38]. Specifically, the CD34+/CD31−/CD45−/CD146− cell subset localized within adventitia, distinct from typical CD34+ endothelial progenitor cells (EPCs), was shown to possess stem/progenitor cell properties and actively participate in vascular pathophysiology [39, 40]. In a vascular injury model, ACs initiated a remodeling process by proliferating and migrating into media and intima and further differentiated into smooth muscle cells, suggesting the importance of adventitia in vascular cell trafficking and blood-vessel remodeling [41, 42]. Furthermore, ACs located in the “vasculogenic zone,” that is, the interface between tunica media and adventitia, have been described as precursors endowed with the capacity to differentiate into endothelial cells and participate in the blood-vessel formation as well as the pathogenesis of atherosclerosis [42–44]. Similar to PCs, there is increasing data suggesting a wide distribution of CD34+ perivascular stromal cells, even at the microvascular level [45].

## 3. Purification of Human Blood-Vessel-Derived Stem Cells

**Figure 1:**
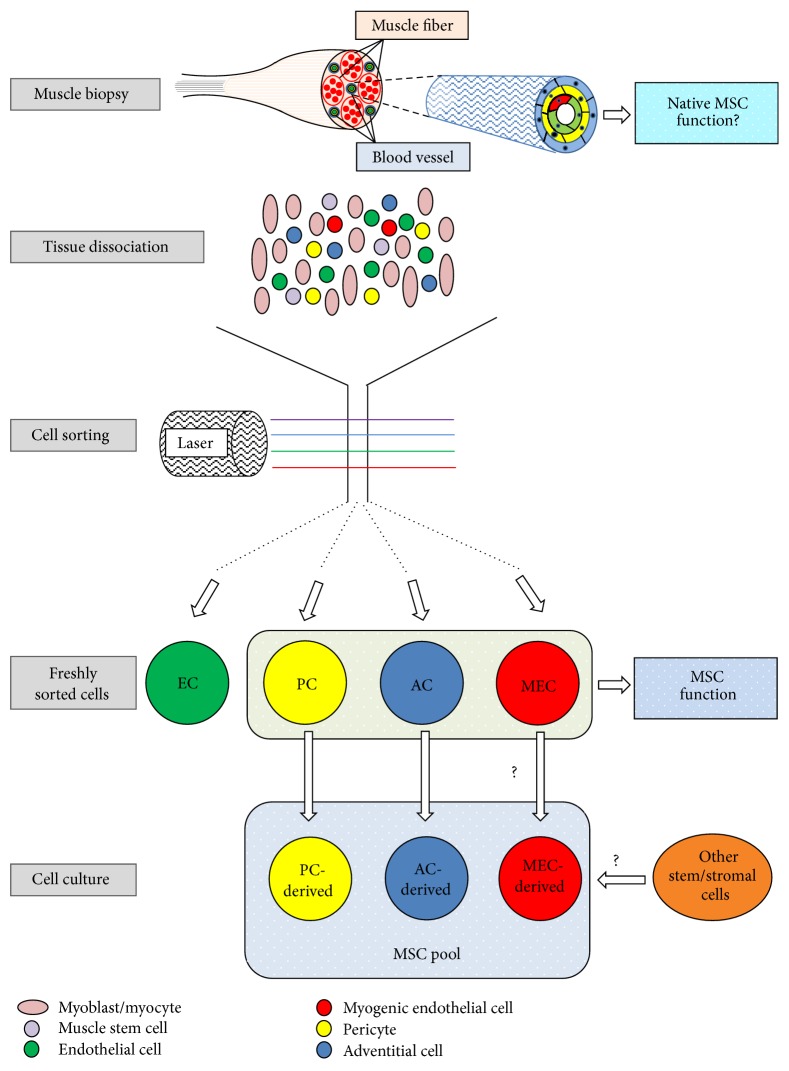
Schematic depiction of hBVSC purification from human skeletal muscle biopsy. Vascular/perivascular cells residing in the blood vessels within the interstitial space of human muscle fibers include endothelial cells (ECs, green), myogenic endothelial cells (MECs, red), pericytes (PCs, yellow), and adventitial cells (ACs, blue). All cells, including immature/mature myocytes and muscle stem cells (i.e., satellite cells), are mechanically and enzymatically dissociated from fresh muscle biopsy. Dissociated cells are subsequently purified to homogeneity by fluorescence-activated cell sorting (FACS). Newly sorted MECs, PCs, and ACs readily exhibit multilineage developmental potentials. Purified PCs, ACs, and possibly MECs give rise to authentic MSCs in long-term culture. However, whether other stem/stromal cells participate in the MSC entity remains to be tested. Moreover, whether native hBVSCs function as typical MSCs* in situ* and/or actively repair/regenerate defective tissues require further investigation.

Based on the cell surface marker expression identified by immunohistochemistry, we discovered a unique combination of surface antigens for each subset of hBVSCs that allows one to purify these cells to homogeneity through FACS: MECs (CD34+/56+/144+/45−), PCs (CD146+/34−/45−/56−), and ACs (CD34+/31−/45−/56−/146−) [16, 18, 28]. The isolation and purification of hBVSC subpopulations have been well established [46]. The workflow of hBVSC purification from fresh human skeletal muscle biopsy is illustrated in Figure 1. To date, skeletal muscle is the only human tissue that has been shown to contain all three hBVSC subsets, with MECs not yet identified in other adult human organs. To isolate PCs and ACs from human adipose, fresh biopsy or lipoaspirate is dissociated mechanically and enzymatically to obtain stromal vascular fraction (SVF), followed by similar cell labeling and sorting processes [16, 18, 47]. PCs can also be purified from human placenta, pancreas, skin, heart, and other organs following a similar protocol [16, 48]. ACs, on the other hand, can be isolated from other human tissues including lung and BM or directly from blood vessels such as saphenous vein [19].

MECs exist at a very low frequency (<0.5%) within the vasculature of human skeletal muscle [28]. On the contrary, PCs can be found in many human tissues at different proportions, for example, 0.29 ± 0.09% in adult skeletal muscle, 0.88 ± 0.18% in fetal skeletal muscle, 0.65 ± 0.10% in adult pancreas, 1.68 ± 0.78% in placenta, and 1.21 ± 0.52% in myocardium [16, 17, 48]. Adipose SVF contains higher frequencies of PCs (14.6 ± 1.02%) due to the abundance of microvasculature in human fat and enrichment of vessel-associated cells during the isolation procedure [16]. On the other hand, ACs represent 9.8 ± 1.7% of the intact adipose SVF and up to 23.8% of SVF from human lipoaspirate for the same reason [18, 47]. In contrast, we found only 2.70 ± 1.01% ACs in human skeletal muscle (unpublished data). Further investigation is needed to determine whether the native frequency of each hBVSC subset changes with multiple physiological parameters, such as age and gender, and/or pathological conditions.

## 4. Characterization of Human Blood-Vessel-Derived Stem Cells

After FACS purification, hBVSCs subpopulations can be either examined/utilized freshly or further expanded in culture [16–18, 47]. MECs, PCs, and ACs have been independently shown to possess mesenchymal differentiation capacities including chondrogenesis, osteogenesis, adipogenesis, and skeletal myogenesis* in vitro* and express classic MSC markers such as CD44, CD73, CD90, and CD105 natively or in culture [16, 18, 28, 36, 49].

MECs not only proliferated at a significantly higher rate than sorted CD56+ MCs and CD34+/144+ ECs, even in low-serum culture conditions, but also were more resistant to cell death when cultivated under oxidative stress (400 *μ*M H_2_O_2_) [28]. PCs, freshly sorted or long-term cultured, from multiple tissues including adipose, placenta, and pancreas exhibited a similar level of skeletal myogenesis* in vitro* as muscle-derived PCs, suggesting a generalized myogenic potential of PCs in the human body [16]. In addition, PCs displayed strong chemotactic response toward papain/pepsin digested ECM harvested from porcine urinary bladder and formed capillary-like structures with/without ECs in two- and three-dimensional Matrigel cultures/cocultures [16, 50]. Similar to PCs, ACs derived from different tissue origins showed the same phenotype and robust mesodermal developmental potentials, suggesting that MSCs can be derived from an alternative systemic source which is distinct from PCs [18]. In regular culture, ACs did not express any of the cultured PC markers including NG2, *α*-SMA, CD146, and PDGFR*β* but shared the expression of vimentin with PCs [18]. The cellular kinetics of hBVSC subsets was recently reviewed in [51].

To further investigate whether hBVSC subsets meet the criteria of* bona fide* stem cells, we obtained clones of MECs, PCs, and ACs through either FACSAria autoclone system or limiting dilutions [16, 18, 28, 49]. At the clonal level, all three hBVSC subsets indeed exhibited typical MSC markers as well as robust mesenchymal differentiation capacities in culture [16, 18, 28, 49]. Consequently we theorized that hBVSC subsets are genuine ancestors of MSCs. Interestingly, it has been shown that ACs proliferate significantly faster than PCs and partially express aforementioned PC markers following treatment of angiopoietins-2 or angiotensin-II, suggesting adaptation of PC phenotypes and/or differentiation into PC-like cells upon stimulation [18]. Therefore it is speculated that ACs serve as the progenitor of pericytes [18, 52]. Nevertheless, future studies with appropriate cell lineage tracking will be required to convincingly establish the hierarchy and developmental relationship among hBVSC subpopulations and between various vascular/perivascular cell populations [53].

## 5. Regenerative Applications of Human Blood-Vessel-Derived Stem Cells

**Figure 2:**
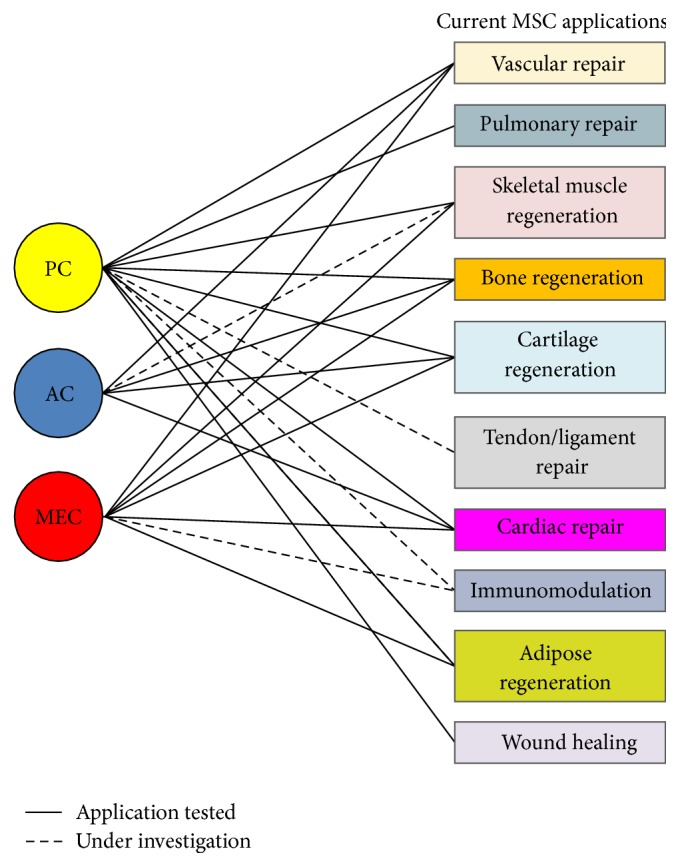
Potential translational applications of hBVSC subpopulations. The current translational applications of typical MSCs are summarized on the right. The current status of translational research for each hBVSC subset is outlined, whether the specific application has been tested or is presently under investigation.

As we described above, each hBVSC subset has been shown to display stem cell characteristics and exhibit mesodermal multipotency upon appropriate induction. In addition, all three hBVSC subpopulations have been demonstrated to serve as robust paracrine units, even under stress conditions [50, 52, 54]. However, the relative significance of paracrine function, direct differentiation, and cellular interaction by individual hBVSC subtype may depend on specific pathological conditions and corresponding regenerative actions.  Figure 2 summarizes putative applications of all three hBVSC subpopulations in regenerative medicine. A number of representative applications will be discussed in detail below.

### 5.1. Skeletal Muscle Regeneration

#### 5.1.1. Myogenic Endothelial Cells

Because of their high myogenic potential* in vitro*, MECs have been tested for muscle regeneration in immunodeficient mouse models of cardiotoxin-induced muscle injury and Duchenne muscular dystrophy [28]. The superior myogenic capacity of MECs over typical CD56+ MCs, CD34+/144+ ECs, and unpurified human primary skeletal muscle cells (hPSMCs) was demonstrated by substantially more regenerating human spectrin-positive myofibers after intramuscular injections into cardiotoxin-injured mouse hindlimb muscles [28]. Moreover, to clarify whether newly regenerated human myofibers originated only from the differentiation/fusion of donor cells or in fact involved the participation of host cells,* in situ* hybridization with mouse X chromosome-specific probe was performed. None of the nuclei within the human spectrin-positive myofibers were identified by the mouse-specific probe, confirming their solely human origin [28]. In addition, regeneration of myofibers coexpressing dystrophin and human lamin A/C was observed in dystrophic mouse muscles after injections of human MECs in mdx/SCID mice [28]. Intriguingly, MECs can also be identified/isolated in nonvertebrates such as leech and directly participate in myogenesis* in vitro* and* in vivo*, similar to their vertebrate counterparts [55]. Recently, the murine counterpart of human MECs was shown to play a role in skeletal muscle homeostasis, inhibiting intramuscular adipogenesis through cell-autonomous and cell-cell interactive mechanisms with active Bmpr1a signaling [56]. Together these data suggest that MECs remain an evolutionarily conserved, distinct myogenic precursor population which actively participates in muscle homeostasis and regeneration and possibly bridges MC and EC during muscle development.

#### 5.1.2. Pericytes

The application of PCs in muscle regeneration was examined in immunodeficient mouse models of cardiotoxin-induced muscle injury and Duchenne muscular dystrophy [16, 17, 36]. Freshly sorted or cultured PCs from human muscle and adipose were injected directly into the cardiotoxin-injured gastrocnemius muscles of SCID/NOD mice [16]. PCs from either tissue source regenerated more human spectrin-positive myofibers than purified MCs or ECs, indicating the authentic myogenic capacity of PCs [16]. Similarly, injections of placental PCs into dystrophic gastrocnemius muscles in SCID/mdx mice not only yielded more dystrophin-positive myofibers but also increased the number of local vWF-positive microvasculatures [17]. The human origin of regenerating myofibers was confirmed by* in situ* hybridization with human-specific probe, coexpression of human lamin A/C, or GFP-based cell tracking [16, 17]. Dellavalle et al. further demonstrated that, through ALP-based cell lineage tracking, native PCs residing in the skeletal muscle participate in the postnatal myofiber development, especially under pathological conditions, and contribute to the satellite cell compartment [37]. These results reflect the robust myogenic potential of PCs* in vivo* that can be generalized to PCs from more clinically accessible nonmuscle tissues like adipose and placenta.

Nevertheless, not all PCs within the skeletal muscle are myogenic and contributing to muscle formation. Birbrair et al. reported the presence of two subtypes of PCs within the skeletal muscle: the adipogenic Nestin−/NG2+ (type-1) and myogenic Nestin+/NG2+ (type-2) PCs [57]. Only type-1 PCs expressed PDGFR*α*, an adipogenic progenitor marker, and contributed exclusively to fat deposition but not myofiber formation after muscle injury [57]. Future research is needed to determine whether successful muscle regeneration depends on a dynamic balance between myogenic type-2 and nonmyogenic type-1 PCs and/or other myogenic and nonmyogenic stem/progenitor cells [58]. Additionally, whether PDGFR*α*+ adipogenic PCs contribute to pathological fat accumulation in myopathies and muscle ageing and their relationship with mesenchymal fibro-adipogenic precursors requires further investigation [59]. These results also imply that separation of subtypes of muscle PCs may be necessary to increase the myogenic efficacy of PC treatment in injured/diseased muscle.

### 5.2. Vascular Regeneration

#### 5.2.1. Myogenic Endothelial Cells

MECs have been shown to form capillary structures in Matrigel culture and implanted Matrigel plugs, suggesting that MECs retain their vascular traits and angiogenic properties* in vitro* and* in vivo*, even after long-term culture [49]. Another vascular progenitor cell (VPC) population coexpressing endothelial and myogenic cell markers (CD34+/133+/KDR+/desmin+) has been derived from human fetal aorta [60]. Upon being transplanted into ischemic muscles, these myogenic VPCs not only alleviated the symptomatic outcome but also incorporated into regenerating myofibers and microvessels in a murine model of hindlimb ischemia [60]. Altogether these studies indicate reparative and regenerative capacities of MECs for angiogenesis and revascularization.

#### 5.2.2. Pericytes

To investigate their capacity for vascular repair, PCs were seeded onto small-diameter, bilayered elastomeric poly(ester-urethane)urea scaffolds and incubated in bioreactors for 2 days before being implanted as aortic interposition grafts in Lewis rats for 8 weeks [61]. PC-seeded grafts showed a significantly higher patency rate (100%) than unseeded controls (38%) and exhibited extensive tissue remodeling including elastin/collagen deposition, multiple layers of *α*-SMA- and calponin-positive cells, and a monolayer of vWF-positive cells in the lumen, indicating the potential of PCs in vascular repair and tissue engineering [61]. Nonetheless, contrary to their typical angiogenic role in tissue repair, PCs were recently demonstrated to inhibit microvessel formation and further induce microvessel dissociation through CXCR3-induced involution of ECs in an* in vitro* angiogenic model [62]. These results suggest multifaceted regulatory functions of PCs in vascular repair/regeneration, not only promoting angiogenesis/vasculogenesis but also contributing to the pruning of excessive/immature microvessels during tissue repair.

#### 5.2.3. Adventitial Cells

The regenerative applications of ACs have primarily been focused on cardiovascular diseases thus far [63]. As described above, ACs actively engage in not only the physiological maintenance but also the pathological remodeling of blood vessels [64]. Consequently, harnessing the restorative power of ACs is key to the success of treating vascular diseases such as atherosclerosis and restenosis of vascular grafts [44, 65].

ACs (CD34+/31−) localized around adventitial vasa vasorum of the human saphenous vein (i.e., adventitial pericytes) have been shown to express typical MSC and certain PC antigens and displayed clonogenic and multilineage differentiation capacities, similar to ACs derived from other tissues [19]. ACs promoted the formation and stabilization of microvessel-like structures* in vitro*, likely through the reciprocal AC-EC interactions and paracrine cross-talk that can be inhibited by Tie-2 or PDGF-BB blockade [19]. Intramuscular injections of ACs in an immunodeficient mouse model of hindlimb ischemia revealed a significant angiogenic effect and facilitated near-full recovery of blood flow by as early as 7 days after injection [19]. ACs remained detectable after 14 days, interacting with host ECs through N-cadherin. These results indicate the potential of ACs in therapeutic angiogenesis/vasculogenesis, especially for the treatment of ischemic diseases. Lately, ACs have been shown to exhibit higher resistance to oxidative stress than ECs due to increased expression of antioxidant enzymes including catalase and superoxide dismutases (SODs) [66]. Silencing the extracellular, soluble isoform of superoxide dismutase (SOD3) in ACs resulted in the negation of their therapeutic benefit on blood flow recovery and neovascularization in a mouse model of peripheral ischemia, suggesting the involvement of SOD3 released by ACs in ischemic protection and/or vascular healing [66].

### 5.3. Cardiac Regeneration

#### 5.3.1. Myogenic Endothelial Cells

The therapeutic potential of MECs in ischemic heart disease was investigated in an immunodeficient mouse model of acute myocardial infarction (AMI) [54]. Myocardial infarction (MI) was first induced by ligating the anterior descending branch of the left coronary artery. Cultured MECs, MCs, and ECs were intramyocardially injected into the ischemic myocardium immediately after the induction of MI [54]. When compared with injections of MCs and ECs, a significant improvement in cardiac contractility was recorded by echocardiography after MEC treatment [54]. Transplanted MECs attenuated ventricular fibrosis, enhanced proliferation and survival of host cardiomyocytes, and promoted local angiogenesis more effectively than MCs and ECs [54]. Despite the robust engraftment of MECs within the infarcted myocardium, only a few differentiated/transdifferentiated into cardiomyocytes [54]. Consequently the functional recovery of MEC-injected hearts resulted primarily from the greater paracrine secretion of trophic factors, especially vascular endothelial growth factor (VEGF), by MECs under hypoxia.

#### 5.3.2. Pericytes

The therapeutic potency of PCs in cardiac regeneration has been investigated. Human muscle-derived PCs were injected into the ischemic myocardium immediately after the induction of MI in a SCID/NOD mouse model [50]. Echocardiography revealed that PC treatment attenuated left ventricular dilatation and significantly improved cardiac contractility, superior to CD56+ MCs [50]. The functional recovery was presumably attributable to the significant increase of host angiogenesis and substantial reduction of ventricular remodeling, myocardial fibrosis, and chronic inflammation at the infarct site [50]. In particular, PCs were shown to have highly active paracrine secretion of trophic factors and cytokines including VEGF-A, PDGF-*β*, TGF-*β*1, IL-6, LIF, COX-2, and HMOX-1, even under hypoxia [50]. In addition to their paracrine function, direct cellular involvement of PCs in cardiac repair was demonstrated by PC homing to perivascular locations and PC-EC interaction* in vitro* and* in vivo* as well as a fraction of PCs differentiating into and/or fusing with cardiac cells [50]. These data suggest that benefits of intramyocardial transplantation of PCs can be attributed to multiple restorative mechanisms involving paracrine effect, cellular interaction, and direct differentiation.

**Figure 3:**
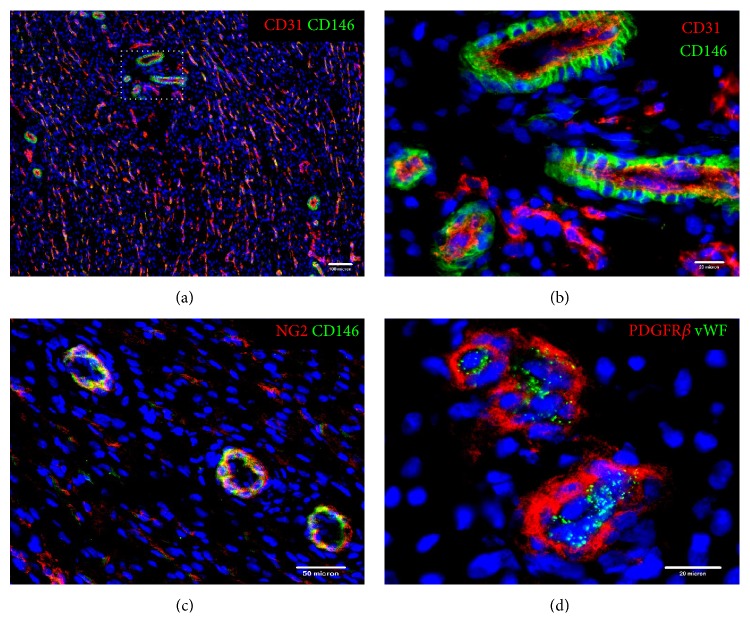
Resident microvascular pericytes in human myocardium. Human myocardium is highly vascularized with numerous microvessels of various sizes and capillaries. Resident heart microvascular pericytes can be identified by a combination of positive (pericyte) and negative (vascular endothelial) cell surface markers. (a) Microvascular and capillary endothelial cells (ECs) were stained by CD31 (red) whilst CD146+ perivascular stromal cells (green) encircled CD31+ ECs (scale bar = 100 *μ*m). (b) Enlargement of the dotted area in (a) further showed that CD146+ human heart pericytes (hHPs, green) closely surround CD31+ ECs (red) (scale bar = 20 *μ*m). (c) CD146+ hHPs coexpressed pericyte marker NG2 (scale bar = 50 *μ*m). (d) hHPs expressing pericyte marker PDGFR*β* (red) encircled vWF+ ECs (green) (scale bar = 20 *μ*m). Nuclei are stained in blue by DAPI (4′,6-diamidino-2-phenylindole).

Very recently, we have successfully identified human heart pericytes (hHPs) based on their surface antigen expression (Figure 3) and purified hHPs by FACS from myocardial biopsies [48]. hHPs (CD146+/CD34−/CD45−/CD56−/CD117−) shared many similarities with their skeletal muscle-derived counterparts and yet showed distinctive antigenic, myogenic, and angiogenic characteristics [48]. Cultured hHPs exhibited typical mesodermal multipotency, except skeletal myogenesis, and displayed prevailing angiogenic reactions under hypoxic conditions when compared with isogenic muscle-derived PCs [48]. Our results suggest developmental and functional divergence of PCs due to anatomical specification. Interestingly, two subpopulations of PCs (Nestin−/NG2+, type-1, and Nestin+/NG2+, type-2) with differential developmental capacities have also been identified within the skeletal muscle [67]. This further suggests that the heterogeneity and developmental divergence of PCs exist not only between PCs from different organs but also among PCs within the same tissue. The tissue-specific signaling and therapeutic potential of hHPs in cardiac regeneration are currently under investigation. Collectively these data suggest that PCs serve as potent regenerative units and growth factor/cytokine sources during tissue regeneration and represent a promising stem cell reservoir, readily accessible throughout the human body, for various therapeutic applications [68].

#### 5.3.3. Adventitial Cells

Katare et al. investigated the efficacy of ACs for treating ischemic heart disease in a mouse MI model [52]. AC treatment improved overall cardiac function and excelled BM-MSC treatment in terms of ameliorating left ventricular dilatation and wall thinning [52]. AC treatment increased local neovascularization, improved myocardial blood flow, and attenuated myocardial fibrosis, cardiomyocyte apoptosis, and vascular permeability [52]. Mechanistically, the paracrine secretion of proangiogenic factors and chemokines presumably activated the proangiogenic and prosurvival Akt/eNOS/Bcl-2 signaling pathway. The involvement of microRNA-132 (miR-132) as a novel paracrine angiogenic stimulant and remodeling inhibitor was demonstrated by blocking miR-132 function in ACs using anti-miR-132, which in turn significantly decreased their vascular supportive capacity* in vitro*, revascularization in the ischemic myocardium, and cardiac reparative/protective functions [52]. Very recently, a combinatory therapy with c-kit+ cardiac stem cells and adventitial pericytes derived from human saphenous vein has been shown to exhibit additive benefits for cardiac regeneration [69]. Together these data strongly support the therapeutic value of ACs in cardiovascular diseases.

### 5.4. Skin Regeneration

#### 5.4.1. Pericytes

The presence of mesenchymal cells, presumably taking on the role of PCs in the healing wound, was first observed in 1970 [70]. The involvement of multipotent dermal PCs in epidermal tissue renewal has been supported by the significantly enhanced regenerative capacity of committed human epidermal cells in organotypic coculture, presumably through the augmented secretion of laminin-*α*5 and independent of angiogenesis, suggesting the importance of pericyte-mediated remodeling of local ECM microenvironment [71]. The role of PCs in wound healing and cell-based wound therapy was reviewed in [35].

### 5.5. Bone Regeneration

#### 5.5.1. Myogenic Endothelial Cells

The potential of MECs for bone regeneration has been examined* in vitro* and in mice [28, 49]. MECs exhibited intense mineralization in pellet culture only with the presence of bone morphogenetic protein- (BMP-) 4, suggesting no spontaneous osteogenic differentiation of MECs without an appropriate inductive signal [49]. *μ*CT imaging revealed that MECs transduced with BMP-4 formed dense ectopic bone nodules when seeded onto a gelatin sponge and implanted into intramuscular pockets in immunodeficient mice [49]. Currently the potency of MEC transplantation for the treatment of critical bone defects is under investigation.

#### 5.5.2. Pericytes

Together with ACs, human adipose-derived PCs have been extensively studied for their bone regenerative capacity [47, 72]. Interestingly, although most demographic parameters, including age, gender, and menopause, did not affect adipose PC yield, donors with body mass index (BMI) less than 25 (nonoverweight) appeared to have higher PC yield than obese donors (BMI > 30) [47]. Moreover, human umbilical cord CD146+ perivascular cells have also been demonstrated as a promising cell source for bone regeneration [73].

#### 5.5.3. Adventitial Cells

Combined with PCs, the skeletal regenerative capacity of human adipose-derived ACs has been explored [47, 72]. Human perivascular stem cells (hPSCs), which comprised only PCs and ACs, were purified from lipoaspirate SVF, seeded onto osteoinductive or control collagen scaffolds, and implanted into either intramuscular ectopic implantation model or critical-sized calvarial bone injury model in immunodeficient mice [47, 72]. When compared with unfractionated SVF, hPSCs formed significantly more bone intramuscularly and led to dramatically greater healing of critical-sized calvarial defects [47, 72]. Additionally, unlike BMP-2 which increased bone formation by hPSCs* in vivo* but also induced an adipogenic response, Nel-like molecule 1 (NELL-1) selectively enhanced osteogenesis of hPSCs and therefore represents a novel osteoinductive growth factor for hPSC-mediated skeletal regeneration [72]. Together recent studies suggest that ACs, like PCs, are functionally superior MSC alternatives for regenerative purposes and effortlessly accessible from dispensable tissues such as lipoaspirate.

## 6. Conclusion

The capability to isolate subpopulations of hBVSCs marked a major progress to understand the heterogeneous MSC entity as well as their vascular/perivascular niches. Purified MECs, PCs, and ACs exhibited robust reparative/regenerative capacities in many injured/defective tissues, often outperforming unfractionated MSCs. No tumorigenesis of any hBVSC subset has been reported thus far, indicating their safety for translational applications. More preclinical studies with large animal models are necessary to further validate the therapeutic safety and efficacy of hBVSCs for clinical use. Currently researchers have planned clinical trials using human pericytes in patients with refractory myocardial ischemia [74]. In addition to fresh tissue biopsies, we have been able to purify MECs and PCs from long-term cryopreserved human primary skeletal muscle cell cultures and further demonstrated their sustained myogenic capacity* in vivo* [75]. This suggests the feasibility to purify specific subset(s) of hBVSCs from either fresh biopsy or banked human primary cells, ultimately facilitating customized regenerative medicine using personalized, homogeneous therapeutic stem/progenitor cell population(s) for a particular pathological condition.
